# Effect of Educational Intervention Based on Theory of Planned Behaviour on Physical Activity Intention among Secondary School Teachers of Nepal

**DOI:** 10.1155/2022/6953632

**Published:** 2022-11-03

**Authors:** Rajan Shrestha, Durga Prasad Pahari, Santoshi Adhikari, Bijay Khatri, Sangita Majhi, Abhinav Vaidya

**Affiliations:** ^1^Academic and Research Department, Hospital for Children Eye ENT and Rehabilitation Services, Bhaktapur, Nepal; ^2^Central Department of Public Health, Tribhuvan University Institute of Medicine, Kathmandu, Nepal; ^3^Nepal Health Research Council, Kathmandu, Nepal; ^4^Department of Community Medicine, Kathmandu Medical College, Kathmandu, Nepal

## Abstract

Any bodily movement produced by skeletal muscle which requires energy expenditure is known as physical activity (PA). WHO has recommended that at least 150 minutes of moderate or 75 minutes of vigorous-intensity PA or a combination of both per week is required for health benefits. Physical inactivity is one of the strongest risk factors for noncommunicable diseases (NCDs) and other conditions and is attributable to 6% of global premature death. However, data on the PA of teachers are unavailable in Nepal. They are considered one of the risk groups for NCDs because of the less active nature of their job. So, we aimed to evaluate the effect of the educational intervention based on the theory of planned behaviour on PA intention among secondary school teachers in Bhaktapur district, Nepal. For this study, we recruited 126 teachers from 6 schools. Each intervention and control group contained three randomly selected secondary schools. All the teachers from the selected schools were enrolled in the respective groups. A quasiexperimental (pretest-posttest control group) study design was used to test the effectiveness of the intervention on attitude, behaviour control, subjective norms, and intention for engaging in regular PA. Both groups underwent baseline and follow-up assessments at four weeks using the self-administered questionnaire developed for this study. The intervention group delivered a one-hour lecture session supported by audio-video materials for PA promotion. The effect was analysed by comparing the changes in the theory of planned behaviour (TPB) constructs within and between intervention and control groups. The difference in scores between and within the groups was tested using Student's *t*-test. Adjusted difference-in-difference scores were calculated through linear regression. Data analysis was done using Statistical Package for Social Science version 26.0. The adjusted mean score increase in TPB constructs due to the interaction of time and intervention increased from 0.641 to 1.381. The highest gain (beta = 1.381) was seen in the intention score, while a minor improvement was seen in perceived behavioural control (beta = 0.641). After the intervention, the net increase in PA intention score was 9.35% compared to the control group. Thus, the promotion package was effective in increasing PA intention. The findings of this study and educational package could be helpful in encouraging teachers to engage in PA in other schools.

## 1. Introduction

Any bodily movement produced by skeletal muscle which requires energy expenditure is known as Physical Activity (PA) [1]. WHO has recommended that at least 150 minutes of moderate or 75 minutes of vigorous-intensity PA or a combination of both is required for health benefits [2]. PA refers to all movement, including during leisure time, for transport to and from places, or as part of a person's work [3].

Noncommunicable diseases (NCDs) such as heart disease, stroke, diabetes, and several cancers can be prevented and managed by regular PA as it helps prevent hypertension, maintains healthy body weight, and improves mental health, quality of life, and well-being [4]. Over the past few decades, physical inactivity has risen to epidemic levels worldwide [5]. Around 30 percent of adults globally do not have sufficient levels of PA [6]. WHO estimates that deaths attributed to noncommunicable diseases (NCDs) in Nepal have risen from 51% in 2010 to 60% in 2014 [7]. A study done on Flemish secondary school teachers in Belgium stated that secondary school teachers' levels of perceived health are low, so they are an important target group for interventions aimed at improving health [8]. In Nepal, the prevalence of low-level PA was high (6%) in the 45-69 age group, and men have higher low-level PA than females [7]. People involved in jobs seemed less physically active in Nepal, and half of the participants believe obesity indicates prosperity [9]; the teaching profession is considered more sedentary, and their PA primarily depends on nonjob-related activities making them among the vulnerable groups for NCDs [10]. In addition, urban residents were associated with lower total self-reported PA in Nepal [11]. Teachers are also considered role models for students and society; physically active teachers can inspire, encourage, guide, and influence others to be active to reduce the burden of insufficient PA [10, 12, 13]. Though information on PA among teachers in Nepal is not yet available, 71.9% of Brazilian teachers have insufficient PA due to the perception of adverse working conditions in teachers, and the study has also recommended the promotion of PA in school teachers [14]. So, we aimed to design a theory of planned behaviour- (TPB-) based educational intervention and test its effectiveness in increasing PA intention among teachers in the public secondary school of Bhaktapur, Nepal.

TPB is a theoretical framework proposed by Ajzen and Fishbein containing attitude, subjective norms, perceived behavioural control (PBC), and intention as the constructs for any health behaviour [15]. TPB is used to design various health interventions, including PA promotion among adults, children, and people with different health conditions [16–19]. Interventions based on this theory were found most effective in increasing PA intention and behaviour than other theories [16, 20, 21], and an increase in PA was also found [22]. There is a link between TPB constructs and change in leisure-time autonomous motivation for PA [23]. PA effect size was also improved using motivating or rewarding approaches [24]. Motivation is a proximal determinant of behaviour, and behaviour change techniques increase motivation in PA [25].

## 2. Materials and Methods

### 2.1. Study Design and Participants

We used a quasiexperimental (pretest-posttest control group) study design to test the effectiveness of the PA promotion package among teachers at public secondary schools in Bhaktapur, Nepal, from January to February 2019. The minimum required sample size was 64, calculated using the intervention study formula *n* = ((*z*_*α*/2_ + *z*_*β*_)^2^*σ*^2^ × 2)/*ϵ*^2^, where *z*_1−*α*/2_ = 1.96 at 95% confidence level, *z*_*β*_ = 0.84 at 80% power of the study, mean desire change in PA intention (*ϵ*) = 0.64, and adding 5% nonresponse rate in calculated sample size [26–28].

Cluster sampling was done to select the municipalities for the study. Among the four municipalities in Bhaktapur district, which have similar local social and cultural contexts, two groups were created from the two municipalities, each located in the eastern and western regions of the district, to avoid contamination. One group was created with the schools of Bhaktapur and Changunarayan Municipalities, having 11 schools with 235 teachers. In contrast, another group was formed with the schools of Madhyapur Thimi and Suryabinayak municipalities, having seven schools with 125 teachers. Then, three schools from each group were randomly picked through the lottery method and assigned as intervention and control groups. The author approached the selected schools through physical visits along with the permission letter from the respective municipality's education section.

### 2.2. Entry Criteria

**Figure 1:**
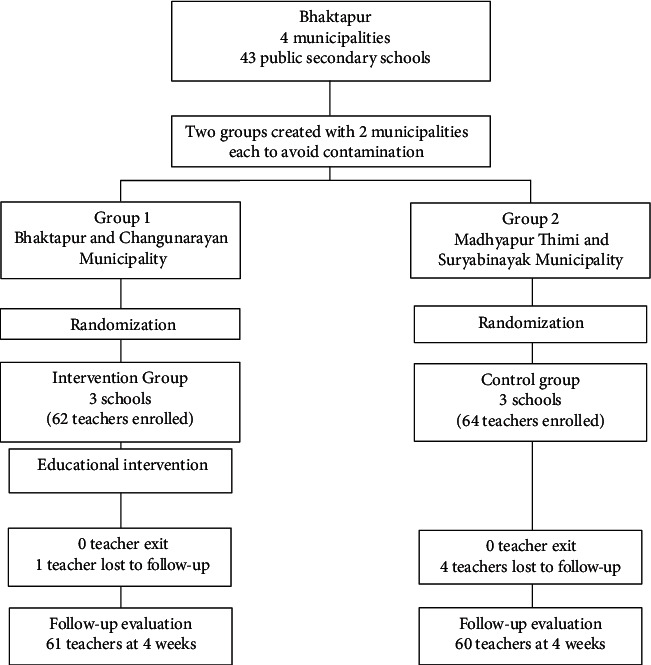
Flow chart of participant enrolment.

All the healthy teachers from the selected schools who consented to participate were recruited in the study using the list of teachers from their respective schools. Finally, the intervention group had 62 teachers, and the control group had 64 teachers. The participants' enrolment is illustrated in Figure 1.

### 2.3. Exclusion and Exit Criteria

The participants absent during the follow-up assessment were excluded from the analysis. There were no exit criteria in this study.

### 2.4. Intervention Description

Ajzen and Fishbein's TPB was used as a theoretical framework for designing a teacher-targeted PA promotion package [15]. Four constructs of TPB, viz., attitude, subjective norms, perceived behavioural control, and behavioural intention towards PA, were considered while designing the promotion package. The PA promotion package was developed using a P-process framework, which has been used to design strategic health communication programs worldwide since 1991 and was improved in 2003 [29]. TPB-based interventions are effective as it has constructs that lead to intention and behaviour [23]. P-process has five steps: analysis, strategic design, development and testing, implementation and monitoring, evaluation, and replanning. We used TPB in the situation, audience analysis, and strategic design of the intervention package. The content of the package mainly promotes indoor PA and adopting PA in the daily schedule they are involved in. The promotion package was also developed by referencing previously published books, booklets, guidelines, videos, and pamphlets. Experts on the topic were also consulted from time to time to validate the promotional package. The item-content validity index is 0.8 (out of 5 experts, 4 rated the content as very relevant) [30].

Similarly, baseline and follow-up assessment questionnaires were set for intervention and control groups using a structured questionnaire prepared, referencing the TPB questionnaire construction example given by Icek Ajzen with modification to the local context [31]. Translation and retranslation of the instruments were done.

Baseline data were collected from intervention and control groups using a structured questionnaire. The intervention was done on the same day after baseline data collection. The package was delivered to the intervention group through a one-hour interactive lecture session, including brainstorming and group discussion; a PA promotion booklet; PA engagement schedule sheets; and motivating short messages sent to the participants' mobile numbers on the second day, first week, and second week of the lecture day. The lecture was given to a group of 15-20 teachers from each intervention school separately by a health promotion graduate in their meeting hall. The intervention and follow-up data collection work was given during the winter season in February 2019. The participants, persons administering the intervention, and persons assessing the outcome were not blinded in this study. After four weeks of intervention, a follow-up assessment of both intervention and control groups was done using the same questionnaire used in the baseline. Then, the control group was also provided with the same intervention after the follow-up assessment. Components and types of PA promoted in the educational intervention package is shown in Table 1.

The details of the intervention package development and contents are attached as supplementary material (available here).

### 2.5. Outcome and Measurement

Knowledge, attitude, subjective norms, behavioural control, and intention for regular PA among the participants were the primary outcomes of this study. Direct measures of the TPB with a 24-item structured questionnaire in a 5-point (range 1 to 5) unipolar Semantic Differential and Likert scale were used for baseline and follow-up to assess those outcomes. Knowledge regarding PA was assessed using 10 knowledge-related questions with one correct answer among 3 options. A follow-up assessment was done after four weeks of intervention. Involvement of at least 150 minutes (at least 30 minutes per day for at least 5 days a week) of moderate-intensity PA with muscle-strengthening exercise at least 2 days a week was considered regular PA [2]. Data was collected in the school setting where teachers work.

## 3. Contents of Knowledge and TPB Constructs

### 3.1. Knowledge

Knowledge constructs have 10 questions with a total score of 10. Each question has 3 responses: 1 (wrong), 2 (right), and 3 (do not know). Examples of the questions are as follows: “PA are the activities done with the skeletal muscle,” “Strolling is a vigorous PA.”

### 3.2. Attitude

This construct has 4 items in a unipolar semantic differential scale with a total of 20 scores. The response ranges from 1 (extremely harmful) to 5 (extremely beneficial) ratings. Cronbach's alpha value on this scale was 0.813. Examples of the questions are as follows: “For me, engaging in regular PA in a forthcoming month is…,” “For me, engaging in regular PA in a forthcoming month is….”

### 3.3. Subjective Norms

This construct has 3 questions with a total score of 15. The Cronbach alpha value of this construct was 0.684. Each question has 1 (strongly agree) to 5 (strongly disagree) rating on the Likert scale. Examples of the questions are as follows: “The people who are important to me think that I should be engaged in regular PA from the forthcoming month,” “The people in my life whose opinions I value, help me to be engaged in regular PA from the forthcoming month.”

### 3.4. Perceived Behavioural Control

This construct has three questions on a unipolar semantic differential scale with 1 (strongly not possible) to 5 (strongly possible). The total score was 15. The Cronbach alpha value of this construct was 0.877. Examples of the questions are as follows: “For me to engage in regular PA in a forthcoming month would be…,” “I am confident that I could be engaged in regular PA from forthcoming month if I wish….”

### 3.5. Intention

This construct has four questions on a Likert scale with ratings of 1 (strongly agree) to 5 (strongly disagree). The total score was 20. The Cronbach alpha value of this construct was 0.688. Examples of the questions are as follows: “I intend to be engaged in regular PA in the forthcoming month,” “I will try to be engaged in regular PA in the forthcoming month.”

## 4. Validity and Reliability of the Tools

### 4.1. Validity

These tools were developed with the consultation of a panel of experts, including physiotherapists, public health experts, clinical psychologists, health promotion and education experts, and school teachers and using the guideline on TPB-based questionnaire development [31, 32]. Study team accepted its face and content validity as per measurement guidelines against the conceptual definition of the construct.

### 4.2. Reliability

Data were collected by a single trained enumerator using the same tools. The tools were pretested in a nonstudy school. Internal consistency of the questions on constructs of TPB with Semantic Differential and Likert scale was assessed using “Cronbach's alpha (*α*).” The value of *α* for each construct was greater than 0.7 in two constructs and 0.68 in another two constructs, which were adequate [33, 34].

### 4.3. Data Analysis

Errors in data entry were minimised by careful data entry and applying checks in EpiData version 3.1, and analysis was done using IBM SPSS Statistics for Windows, Version 26.0 (Released 2019. IBM Corp., Armonk, New York, United States). The normality of the data was tested, and a parametric test was applied for data analysis accordingly. The difference in baseline and follow-up knowledge and TPB construct scores of individual teachers between and within intervention and control groups was compared by applying Student's *t*-test. Unadjusted difference-in-difference (DiD) was calculated by subtracting the change in score among the control group (time effect) from the change in score among the intervention group (effect of time and intervention). Regression modelling was done using the equation *Y* = *β*_0_ + *β*_1_^∗^ [time] + *β*_2_^∗^ [intervention] + *β*_3_[time^∗^ intervention] + *β*_4_[covariates] + *ε* to calculate adjusted interaction between time and intervention, where *Y* is the total mean score and *β*_3_ is difference-in-difference (DiD) [35].

### 4.4. Ethical Consideration

Ethical approval was taken from the Institutional Review Committee of the Institute of Medicine, Tribhuvan University (Ref no. 45(6-11.E)2/075/076). Permission was granted from municipalities and school authorities. Participation was voluntary, and written informed consent from the participants was obtained before enrolling them in the study.

## 5. Results

Among 62 and 64 participants enrolled in the intervention and control groups, respectively, one (1.61%) teacher in the intervention group and four (6.25%) in the control group dropped out due to their absence during follow-up. The participants' sociodemographic, teaching, PA, and health-related characteristics in both arms are not statistically significantly different (Table 2).

The mean follow-up score in all TPB constructs, including the knowledge, increased among the intervention group. In contrast, the scores were slightly decreased among the control group in all TPB constructs except the intention score. The unadjusted DiD mean scores ranged from 0.62 to 1.38 points, raised by 5.32%-10.22%. The maximum unadjusted DiD was seen in the knowledge score (10.22%). The unadjusted DiD point score was seen in intention (1.38), which increased by 9.35% from the baseline score in the intervention group (Table 3).

The adjusted increase in mean score due to the interaction of time and intervention increased from 0.641 to 1.381. The highest gain (beta = 1.381) was seen in the intention score, while the minor improvement was in perceived behavioural control (beta = 0.641) (Table 4).

## 6. Discussion

This study tested the TPB-based promotion package with the intention of engaging in regular PA among school teachers of the Bhaktapur district, Nepal. Our intervention was a part of a study that also assessed the status of PA, its correlates, barriers, and facilitators of engaging in regular PA, which will be reported in a separate paper. TPB was used as a theoretical framework by many studies on PA [28, 36–38] and found effective in increasing PA intention [28].

The adjusted mean score increase in TPB constructs due to the interaction of time and intervention increased from 0.641 to 1.381. The highest gain (beta = 1.381) was seen in the intention score, while a minor improvement was seen in perceived behavioural control (beta = 0.641). After the intervention, the net increase in PA intention score was 9.35% compared to the control group.

The findings of this study have also shown that the package effectively increased the mean scores in all four constructs of TPB and knowledge.

Regarding TPB constructs, change in mean score in follow-up from baseline on knowledge (0.82), attitude (1.3), subjective norms (0.66), perceived behavioural control (0.62), and intention (1.38) on PA showed a larger increment in the intervention group than in the control group. Within the group, baseline and follow-up score difference was significant in all constructs except for subjective norms and perceived behaviour control. This may be due to the focus of the intervention package on motivating the participant; it is not adequate to change the perceived influence of the people who can influence the participant to be physically active. The change in the control group could be the time effect, while the difference in the intervention group could be due to the time effect and intervention. While looking at the difference in mean point score, positive change is seen in the intervention group in all constructs. Surprisingly, the difference in the control group is slightly negative except for the intention score, which was also seen in a study done by White et al. among people with diabetes and cardiovascular disease [39]. This may be due to the cold winter during the study period with increased outdoor air pollution, being an unfavourable environment for being physically active [40]. Despite the improvement seen in all constructs due to time and intervention interaction, *R*^2^ values range from 0.034 to 0.123 which indicates that only 3.4%-12.3% changes is explained by the model.

TPB-based interventions on the PA done by researchers used various interventions targeted to specific groups with different purposes and duration. Our intervention was designed to increase the four constructs of TPB among apparently healthy school teachers through an interactive lecture with the addition of participants' engagement in their PA planning and adding action cues to increase the effect of the intervention, as one session of the one-hour lecture may not be sufficient in increasing TPB constructs. We selected school teachers as they are less physically active and would be there in schools for follow-up assessments [14, 41]. A study done by White et al. used extended TPB, which also included planning and PA behaviour, added to a 2-hour session delivered weekly over four weeks among type 2 diabetes and cardiovascular diseases and tested the effect in one and six weeks [39]. We mainly focused on promoting an active lifestyle that could be achieved through minor modifications in daily activities that teachers readily accepted. This may be the reason for the positive impact on all TPB constructs.

In another study, one-hour intervention in seven sessions for obese military personnel was given to increase PA and tested the effect in 3 months, had improved all constructs of TPB in the follow-up than in baseline (*p* < 0.001), while there was no significant change in the control group [42]. In comparison to this intervention, our intervention only significantly changes the control group's attitude, intention, and knowledge scores (*p* < 0.05). A more extended period of follow-up and intervention targeted at people with a particular health condition may increase the impact of the intervention. The intervention with multiple sessions and evaluation after eight weeks showed a significant effect of intervention and time, though the effect size was not calculated [19].

Knowledge was the most improved score with a more than 10% increment. Knowledge is the first step to changing other constructs [43]. This short-term knowledge gain as the follow-up is made in 4 weeks. Likewise, being teachers as study participants, this could also be due to their exposure to other sources of information during the study period, which is beyond the researcher's control.

After the intervention, the attitude score for regular PA within the intervention group was significantly increased in this study, consistent with the findings of another TPB-based intervention done on prediabetic women [44]. This showed that the intervention reinforced the beliefs and attitudes of secondary school teachers, which further contributed to school teachers having a higher tendency to perform PA. This might be because they perceived engaging in PA have more advantages than disadvantages. In a randomised control trial done among patients with type 2 diabetes in Iran, intervention with one session weekly of 2-hour group-based classes delivered by a nutritionist for four weeks also significantly increased intention and attitude scores in 8 weeks (*F* = 4.01, *p* < 0.001, partial *n*^2^ = 0.35) [19].

While there was no statistically significant difference between groups on baseline scores before the intervention, all TPB components except for subjective norms improved significantly (*p* < 0.05) in the intervention group compared to the control group using Student's *t*-test. This indicates that study participants had more expectations from those who were important to help them to be physically active. This construct is related to the people other than the teachers who got intervention; this may be another reason for not improving the score of this construct.

Perceived behavioural control after intervention in this study was not significantly improved as in another study [44]. No improvement in perceived behavioural control showed that the school teachers did not consider themselves more capable of doing PA. This could be attributed to several reasons: no direct intervention based on this construct was provided. Secondly, the follow-up visits may have been insufficient for the measurable changes. At the same time, other studies showed the changes in the perceived behavioural control and intention construct of TPB more remarkable than changes in the different constructs (*p* < 0.01 vs. *p* < 0.05) [42]. The intention is moderately correlated with self-reported behaviour in a week among the diabetic population [39], while 59-61% of intention was turned into behaviour [17].

One of the strengths of this study is the theory-based teacher-targeted PA intervention package which primarily promotes indoor PA to avoid outdoor cold and air pollution, which will be doable and sustainable even during winter. Another strength is the use of TPB as the framework of this study which was widely used for the PA intervention study that could be used to increase PA intention among school teachers. We also used the P-process while designing the educational materials.

This study had some limitations to report. We used a quasiexperimental study design without true randomisation, which may have led to selection bias. The most important limitation in this study was the short duration of follow-up assessment. This emphasises the need for further studies with longer follow-ups. This study evaluated PA intention instead of assessing its behaviour, which is another limitation.

## 7. Conclusions

The PA promotion package effectively improved knowledge, attitude, perceived behaviour control, and intention on PA among the intervention group, significantly different from the control group. But it could not improve subjective norms on PA seriously. The intervention package's net increase in behavioural intention towards PA was 9%. The finding of this study and developed promotion package could be utilised to motivate teachers in other schools to decrease the burden of low-level PA. Future studies should focus on developing community-based PA motivation packages targeted at people with insufficient PA and test their effectiveness on the long-term impact on promoting PA behaviour and its maintenance. PA promotion programs should also become an integral part of the management of NCDs.

## Figures and Tables

**Table 1:** Components and types of PA promoted in educational intervention package.

Component	Content	Method	Media
	Welcome, introduction, and objective of the session	Lecture	PowerPoint slides
Knowledge change about PA	Introduction about PA	Mini lecture brain storming	PowerPoint slides, booklet
Attitude change towards PA	Benefit and risk related with PA	Mini lecture brain storming	PowerPoint slides, booklet
Subjective norms change towards PA	Importance of friends and family in PA	Mini lecture group discussion	PowerPoint slides, booklet
Perceived behaviour control change towards PA	How people with disability are involved in PA, pictures of physically active personality, presenter's involvement in PA	Brainstorming mini lecture	PowerPoint slides, booklet video show, photo show, PA schedule preparation
Types of physical activities promoted in the package	Skipping, push-up, pull-up, cycling, brisk walking, swimming, treadmill, football, volleyball, table tennis, badminton, playing with children, walking with friends/family, working in garden, kitchen garden, use of stairs instead of lift, climbing up and down during leisure periods		

**Table 2:** Baseline characteristics of the participants.

Characteristics		Intervention group	Control group	*χ* ^2^ *p* value
		*n* (%)	*n* (%)	
Sociodemographic characteristics				

Gender	Male	31 (50.0)	27 (42.2)	0.379
	Female	31 (50.0)	37 (57.8)	

Age category (years)	15-29	7 (11.3)	5 (7.8)	0.749
	30-44	28 (45.2)	28 (43.8)	
	45-65	27 (43.5)	31 (48.4)	

Marital status	Married	54 (87.1)	60 (93.8)	0.203
	Unmarried	8 (12.9)	4 (6.3)	

Family type	Joint/extended	34 (54.8)	29 (45.3)	0.285
	Nuclear	28 (45.2)	35 (54.7)	

Caste	Brahmin/Chhetri	22 (35.5)	34 (53.1)	0.105
	Janajati	37 (59.7)	29 (45.3)	
	Ethnic minority	3 (4.8)	1 (1.6)	

Educational level	Up to bachelor	33 (53.2)	41 (64.1)	0.217
	Master and above	29 (46.8)	23 (35.9)	

Teaching-related characteristics				

Teaching level	Basic	36 (58.1)	43 (67.2)	0.290
	Secondary	26 (41.9)	21 (32.8)	

Teaching experience (years)	Up to 10	15 (24.2)	18 (28.1)	0.671
	11-20	23 (37.1)	19 (29.7)	
	20+	24 (38.7)	27 (42.2)	

Teaching health subject	Yes	7 (11.3)	5 (7.8)	0.506
	No	55 (88.7)	59 (92.2)	

PA-related characteristics				

WHO recommendation for PA	Not meet	8 (12.9)	8 (12.5)	0.946
	Meet	54 (87.1)	56 (87.5)	

PA level	Moderate to high	52 (83.9)	53 (82.8)	0.873
	Low	10 (16.1)	11 (17.2)	

Health-related characteristics				

Raised blood pressure	No	47 (75.8)	46 (71.9)	0.616
	Yes	15 (24.2)	18 (28.1)	

BMI category	<25	16 (25.8)	20 (31.3)	0.499
	≥25	46 (74.2)	44 (68.8)	

WHtR category	<0.5	9 (14.5)	8 (12.5)	0.741
	≥0.5	53 (85.5)	56 (87.5)	

Raised blood pressure = SBP ≥ 90 mmHg or DBP ≥ 140 mmHg.

**Table 3:** TPB constructs at baseline and follow-up among intervention and control groups.

Variables	Group	Baseline mean (SD)	Follow-up mean (SD)	*p* value	Mean score difference baseline to follow-up	The difference-in-difference (improved %)
Knowledge	Control	8.02 (1.35)	7.93 (1.22)	0.726	-0.09	
	Intervention	8.02 (1.36)	8.75 (1.30)	0.001	0.73	0.82 (10.22)

Attitude	Control	16.73 (2.03)	16.54 (2.27)	0.616	-0.19	
	Intervention	16.06 (2.55)	17.17 (2.34)	0.014	1.11	1.3 (8.09)

Subjective norms	Control	11.66 (1.83)	11.52 (1.85)	0.690	-0.14	
	Intervention	10.90 (1.59)	11.42 (1.36)	0.058	0.52	0.66 (6.06)

Perceived behavioural control	Control	12.14 (1.40)	11.89 (1.76)	0.370	-0.25	
	Intervention	11.66 (1.67)	12.03 (1.54)	0.204	0.37	0.62 (5.32)

Intention	Control	15.23 (1.72)	15.59 (1.63)	0.237	0.36	
	Intervention	14.76 (2.41)	16.50 (1.78)	<0.001	1.74	1.38 (9.35)

**Table 4:** Effect of intervention (difference-in-difference) on different TPB constructs on PA.

	Knowledge	Attitude	Subjective norms	Perceived behavioural control	Intention
	Beta (95% CI)	Beta (95% CI)	Beta (95% CI)	Beta (95% CI)	Beta (95% CI)
Constant	7.79 (7.19-8.40)	16.74 (15.69-17.88)	11.43 (10.62-12.25)	11.79 (11.00-12.57)	15.13 (14.20-16.05)
Intervention	-0.02 (-0.46-0.41)	-0.68 (-1.50-0.13)	-0.77^∗^ (-1.36-(-0.19))	-0.45 (-1.02-0.12)	-0.50 (-0.17-0.16)
Time (follow-up)	-0.08 (-0.52-0.35)	-0.19 (-1.01-0.62)	-0.14 (-0.72-0.45)	-0.25 (-0.82-0.31)	0.36 (-0.31-1.02)
Time (follow-up) and intervention	0.82^∗^ (0.19-1.44)	1.31 (0.14-2.47)^∗^	0.66 (-0.17-1.49)	0.64 (-0.16-1.44)	1.38^∗^ (0.44-2.33)
Age 40 years and above	-0.47^∗^ (-0.78-(-0.16))	-0.07 (-0.66-0.52)	-0.22 (-0.65-0.19)	0.33 (-0.07-0.74)	-0.59^∗^ (-1.07-(-0.11))
Married	0.71^∗^ (0.16-1.27)	0.36 (-0.68-0.47)	0.91^∗^ (0.17-1.65)	0.42 (-0.30-1.13)	0.41 (-0.44-1.25)
Education bachelor and above	0.06 (-0.26-0.37)	-0.12 (-0.71-0.47)	-0.17 (-0.59-0.25)	-0.02 (-0.43-0.38)	0.36 (-0.12-0.84)
Teaching experience 10 yrs. and above	-0.26 (-0.63-0.11)	-0.31 (-1.01-0.39)	-0.53^∗^ (-1.03-(-0.03))	-0.27 (-0.75-0.21)	-0.14 (-0.71-0.43)
*R* ^2^	0.123	0.034	0.066	0.031	0.138

^∗^Significant at *α* < 0.05.

## Data Availability

The datasets used and/or analysed during the current study are available from the corresponding author on reasonable request.

## Supplementary Materials

Supplementary Materials Table S1: summary of intervention package.
