# Supplementary material for: Effect of Educational Intervention Based on Theory of Planned Behaviour on Physical Activity Intention among Secondary School Teachers of Nepal
Source: Biomed Res Int. 2022 Nov 3;2022:6953632. doi: 10.1155/2022/6953632 (PMC9649301; doi:10.1155/2022/6953632)
Supplement: Supplementary Materials — Table S1: summary of intervention package. [file 6953632.f1.docx]

**Supplementary materials:**

**Teacher-targeted physical activity promotional package development**

Educational diagnosis based on PRECEDE model was used in assessing factors associated with physical activity. Reinforcing and enabling factors related to physical activity were adopted from the model. The theoretical framework used was adapted from Ajzen and Fishbein’s Theory of Planned Behavior (TPB).^15^ Four constructs of TPB, viz. attitude, subjective norms, perceived behavioral control, and behavioral intention towards physical activity, were used while designing and evaluating the intervention package.

The physical activity promotion package was developed based on the steps of the P-process. The p-process is a framework designed to guide communication professionals in developing strategic communication programs. It has been used successfully worldwide to create health communication programs since 1982.^29^

**Promotion package development:** A promotional package for physical activity development was done. The information obtained from the situation analysis study was used, and formative research using focus group discussion was conducted. The package development process was done following P-process with the following steps:

1. **Analysis:**

*Situation Analysis:* Situation analysis was done with the findings of the situation analysis study. Four focus group discussion was also conducted to explore the perception, barriers to physical activity, and how those barriers could be overcome.

*Audience Analysis:* The background characteristics and physical activity status were used in the situation analysis study. The appropriate method and media were assessed through FGD.

**Findings of formative study used during the development of the promotional package**

**Perceptions about regular physical activity:** Most participants perceived physical activity as everyday work from early morning to late night. Physical activity makes the body healthy. Half of them perceived it as doing yoga, aerobic exercise, walking, cycling, etc. Some participants felt it is needed after 40 to be healthy. A male teacher said, "*I think physical activities are activities we do every day, from early morning to late night.*"

**Perception about the adequacy of physical activity:** Most participants perceived they did not have enough physical activity. A female participant said, "I am swamped *doing household work, though my weight is not under control*."

**Barriers to physical activity:** Most participants feel their laziness, lack of willpower and less priority for physical activity were the main barriers to being active. About half of the participants were busy teaching from early morning to late night and did not get enough physical activity. Most of the female participants were alive with light household chores. Another barrier is the bike or scooter they use for travel which decreases walking. Some participants fear the adverse effect of outdoor air pollution. A female participant felt her mother-in-law was unsupportive. Some participants think they do not have friends and family members who encourage them to be physically active. A female teacher said, "*I need to fight with my mother-in-law to go for a morning walk*."

**A possible solution to overcome barriers:** Most participants feel self-motivation and power will be the best solution to be physically active. Some participants said it would be happening if they had busy friends. Half of the participants feel they will be active if it is mandatory. A male teacher said, "*It is all about willpower that could help me be involved in regular physical activity; I can walk to school and reduce the use of mobile but not motivated enough to do so."*

1. **Strategic design**: The theory of planned behavior was used to develop and implement the intervention package. A promotional package for physical activity, including objectives of the education package, target groups, the content outline, methods, media, implementation plan, and evaluation plan, was prepared. The details of the strategic design are included in Annex III.
2. **Development and testing:** After the strategic design, the promotional package for physical activity was developed. The package contains a mini-lecture with a PowerPoint presentation, booklet, and physical activity schedule sheets. The evaluation tool was also developed based on the theory of planned behavior. While developing the evaluation tool and package, constructing a TPB Questionnaire manual, existing physical activity promotional material was reviewed, and expert consultation (physiotherapist, public health physician, clinical psychologist) was also done.

The developed package was pre-tested among teachers at a public secondary school in Chapagaun, Godawari Municipality, Nepal. The contents of the box were delivered a day before the pretest. A mini-lecture session with a PowerPoint presentation, booklet, and physical activity schedule sheet was done. The pretesting was done to determine whether the materials and message were understandable to the target audience, believable or not, and culturally acceptable. The purpose of the pretesting was to assess whether the package motivated the audience to change behavior and to get the necessary suggestion to improve the package.

1. **Implementation:** Implementation of the promotion package was carried out based on the developed implementation plan of the package. The intervention was given in three schools (two from Bhaktapur municipality and one from Changunarayan municipality) with the participation of 62 teachers. Mini-lecture sessions using PowerPoint presentations in Nepali, brainstorming, and group discussion were carried out. The researcher himself carried out all the sessions.

**Table S1: Summary of Intervention package**

| Area of Intervention | Engaging in regular physical activity |
| --- | --- |
| Priority audience | Teachers at public secondary school |
| Objectives | The objective of the promotion package was to develop intention toward physical activity by teachers of the public secondary school in Bhaktapur district. |
| Methods | Interactive Mini-Lecture with a PowerPoint presentation in Nepali, Video show, Group discussion |
| Media | - Visual Media   - Infographics, Fat to fit personality picture, researcher's picture of the involvement of physical activity   - PA motivating guide booklet developed (8 pages, A4 Size one color, glossy paper, 200 booklets) - Audio Visual media   - WHO physical activity motivating video, videos of involvement of people with a physical disability |
| Duration | 1 hour in each school |
| Resource person | Researcher himself (Mr. Rajan Shrestha) |
| Participants | Group of 13 to 30 teachers |
| Cues to Action | It is a motivating SMS sent to each participant on the second day, one week, and two weeks after the session. |
| Contents outline of the package | - Introduction to physical activity, its type, and why physical inactivity is a problem? Concept of energy balance, the idea of Frequency, Intensity, Time, and Type of physical activity - Recommended level of physical activity - Benefit of regular physical activity. Indicators to monitor - Blood Pressure, BMI, Waist to height ratio, Tips for engaging in physical activity, Physical activity pyramid - physical activity goal setting, scheduling, physician consultation and precautions, the concept of brisk walking, climbing ladder, Zumba dance, and indoor walking exercise |

**Objectives:** The promotion package aims to develop intention toward physical activity by teachers at the public secondary school of Bhaktapur district.

**Target group:** Teachers of the public secondary school in Bhaktapur district

**Methods and media of education**

**Methods:** Interactive Mini lecture with a PowerPoint presentation in Nepali, Video show, and group discussion

**Media**:

- Visual Media
- Infographics, Fat to fit personality picture, researcher's image of the involvement of physical activity
- PA motivating guide booklet developed (8 pages, A4 Size one color, glossy paper, 200 booklets)
- Audio-Visual media
- WHO physical activity motivating video from <https://youtu.be/uZX14W4rVCU>, videos of involvement of people with physical disability <https://youtu.be/554lkaSNQh4>, Zumba dance from <https://youtu.be/YxWgmhp0_9w> and Walking at home exercise from <https://youtu.be/-SSYX8sIOmM>
- Cues to Action: Motivating SMS sent to each participant second day, one week, and two weeks after the session.

**Duration**: 1-hour session

**Action cues**: Reminder for engaging in physical activity, including short motivational messages sent to mobile phones on the second day, the first week, and the second week

**The second day after the session:**

“Have you made your weekly physical activity schedule sheet and started engaging in physical activity for at least 30 minutes daily? Please do moderate intensity slowly at the beginning.”

**After a week of sessions conducted:**

“Being physically active will reduce the risk of heart disease, blood pressure, diabetes, and cancers, saving your money and time. Have you engaged in physical activity today? Active life, healthy life, happy family.”

**After two weeks of sessions conducted:**

“30 minutes investment in regular physical activity for a healthy life”

**Contents outlines of the package:**

- Introduction to physical activity, its type
- Why is physical inactivity a problem?
- Concept of energy balance
- concept of Frequency, Intensity, Time, and Type of physical activity
- Recommended level of physical activity
- The benefit of regular physical activity
- Indicators to monitor - Blood Pressure, BMI, Waist to height ratio
- Tips for engaging in physical activity
- Physical activity pyramid
- physical activity goal setting, scheduling
- physician consultation and precautions
- concept of brisk walking, climbing ladder, Zumba dance, and indoor walking exercise
- pictures of physical activity shown
- photo of fat to fit celebrity used

**Validity of the package:**

The situation analysis study and Focus Group Discussion were used to validate the physical activity promotion package. The package was developed by reviewing the existing material available. The theory of Planned Behavior was used as a theoretical framework to design and create the content of the package. Experts on Physical activity research (Community Health Physician with PhD Degree, Clinical Psychologist, Physiotherapist) was consulted to validate the package.

1. **Evaluation:** The effectiveness of the package was assessed by conducting a quasi-experimental pretest-posttest control group design. The effectiveness of the package was measured in terms of an increase in knowledge, attitude, subjective norms, perceived behavioral control, and intention towards physical activity**.**
